# Effect of IL-2 co-expressed or co-inoculated with immuno-dominant epitopes from VP1 protein of FMD virus on immune responses in BALB/c mice

**DOI:** 10.22038/ijbms.2019.31972.7683

**Published:** 2019-03

**Authors:** Mohammad Doosti, Mohammadreza Nassiri, Khadijeh Nasiri, Mojtaba Tahmoorespur, Saeed Zibaee

**Affiliations:** 1 Department of Animal Science, Ferdowsi University of Mashhad, Mashhad, Iran; 2 Recombinant Proteins Research Group, Research Institute of Biotechnology, Ferdowsi University of Mashhad, Mashhad, Iran; 3 Department of Exercise Physiology, Faculty of Sport Science, University of Mazandaran, Babolsar, Iran; 4 Razi Vaccine and Serum Research Institute, Mashhad, Iran

**Keywords:** Adjuvant, Foot-and-mouth disease-virus, Immune response Interleukin-2, VP1 protein

## Abstract

**Objective(s):**

The results of studies on vaccine development for foot-and-mouth disease (FMD) virus show that the use of inactivated vaccines for FMD virus is not completely effective. Novel vaccinations based on immuno-dominant epitopes have been shown to induce immune responses. Furthermore, for safety of immunization, access to efficient adjuvants against FMD virus seems to be critical.

**Materials and Methods:**

In this study, we produced epitope recombinant vaccines from the VP1 protein of the FMD virus for serotype O of Iran. Constructs were included polytope (tandem-repeat multiple-epitope), polytope coupled with interleukin-2 (polytope-IL 2) as a molecular adjuvant and IL-2. Three expression vectors were constructed and expressed in *Escherichia coli* BL21 (DE3). To evaluate whether these recombinant vaccines induce immune responses, BALB/c mice were injected with the recombinant vaccines and their immune responses were compared with a negative control group. The humoral and cellular immune responses were measured by ELISA.

**Results:**

The results showed that IL-2 co-expressed or co-inoculated with Polytope protein enhances the immune effect of multiple epitope recombinant vaccine against FMD virus. The results of total immunoglobulin G (IgG), IgG1, and IgG2a levels and secretion of interferon gamma (IFN-γ), IL-4 and IL-10 revealed that there were significant differences between negative control group and other injected mice with the recombinant vaccines (*P<*0.05).

**Conclusion:**

Observations indicated that the epitope recombinant plasmid of the VP1 protein co-expressed or co-inoculated with IL-2 was effective in inducing an enhanced immune response. Therefore, IL-2 can be recommended as a potential adjuvant for epitope recombinant vaccine of the VP1 protein from FMD virus.

## Introduction

Food and mouth disease (FMD) is a common viral disease in cloven-hoofed animal such as cattle, buffalo, sheep, goats and pigs. This disease is distinguished with fever and bullous lesion on the tongue and lips in the mouth, under the breast and between hoof, resulting in weight loss, reduced milk production and growth delays. FMD virus leads to remarkable economic losses in livestock industry wherein cumulative negative effects of this disease have impact on animal production. FMD virus is one of the main obstacles to provide animal health and animal production (1). 

To date, seven serotypes of FMD virus have been identified, including A, C, O, Asia 1, SAT1, SAT2 and SAT3. Different variants and many subtypes exist within the seven subtypes. FMD is a member of the genus Apthovirus of the Piconaviridae family that the viron contains a positive sense, single copy of 8.5 kbp RNA genome wrapped with 60 copies of each of VP1, VP2, VP3 and VP4 proteins (2). Studies showed that the VP1 protein determines the serotype of FMD virus and is one of the components responsible for induction of neutralizing antibodies (3, 4).

Traditional FMD virus vaccines have some disadvantages such as necessity of revaccination, and problems in distinguishing infected animals from vaccinated animals. The serotypes of FMD virus have immunogenic difference that makes it necessary to have serologic matches for the formulation of effective vaccines (5). Also, there is a potential risk of the recovery of live virus during vaccine production (6). On the other hand, previous studies revealed that inactivated vaccines against FMD virus induce neutralization antibodies (7, 8). Nevertheless, the inactivated vaccines pass through the process of the culturing of live viruses during their production (9). For these reasons, to control FMD disease it is necessary to replace new-type vaccines that do not require live virus material. 

Immunological studies of FMD virus showed that the amino acids in the lobe G-H of structural protein VP1 are the main dominant epitopes responsible for the induction of neutralizing antibody responses. Two immunogenic sites on the VP1 protein in O and A serotypes were located at residues 141-160 amino acids and 200-213 amino acids (10, 11). 

As mentioned above, the development of safe recombinant vaccines play a protective role against FMD virus. However, practical applications of candidate recombinant vaccines against FMD virus are limited. Hence, applying appropriate adjuvants to stimulate and enhance immune response to FMD virus seems critical (12, 13). Based on several studies, some cytokines were identified as effective adjuvants of DNA vaccines and cause increasing the induction of immune system (14). Interleukin-2 (IL-2), known as molecular adjuvant, is reported to stimulate the proliferation and differentiation of effector T cells (15).

The objective of the present study was to evaluate the impact of IL-2 as adjuvant co-expressed or co-inoculated with immuno-dominant epitopes of VP1 protein of FMD virus on humoral and cellular immune responses in BALB/c mice.

## Materials and Methods

***Design and synthesis of multiple-epitope gene from FMD virus***

**Figure 1:**
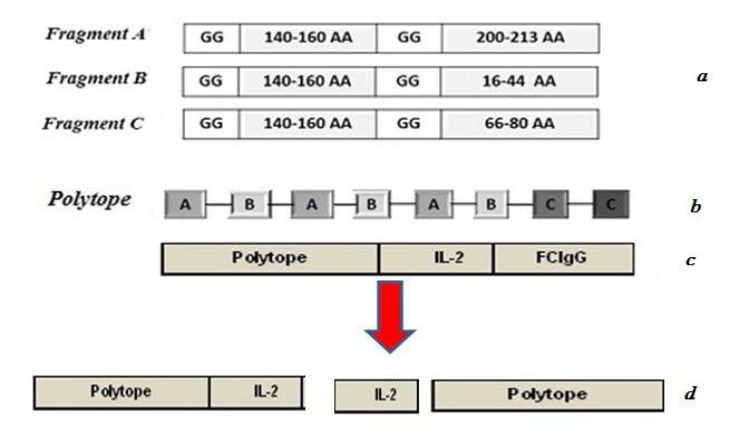
(a) Immuno-dominant epitopes of the VP1 protein of foot-and-mouth disease (FMD) virus. (b) A tandem-repeat multiple-epitope gene (polytope). (c) Construction of the Polytope/interleukin-2 (IL-2)/FcIgG synthetic gene. (d) Gene fragments isolated from the polytope-IL-2-FcIgG fusion gene that were used to construct recombinant vaccines

In our previous studies, the Polytope-IL2-FcIgG fusion gene was synthesized and cloned into the pUC57 vector by Gene Script Company (US) (16). For constructing polytope-IL2-FcIgG gene, four immunogenic epitopes corresponding to amino acid residues 140 to160 (B-cell epitope), 200 to 213 (B-cell epitope), 16 to 44 (T-cell epitope) and 66 to 80 (T-cell epitope) from the VP1 capsid protein of FMD virus for serotype O isolated from Iran (GenBank accession no. HQ663879) were selected. These antigenic epitopes have been reported in previous studies (12, 13, 17). Mouse IL-2 (GenBank accession no. NM-008366.3) and FC fragment of the mouse immunoglobulin (Ig) gamma (CHO_2_–CHO_3_) (GenBank accession no.AAB59660.1) were selected as molecular adjuvants to design and construct a synthetic fusion gene. According to the selected epitopes of the VP1 protein, a tandem-repeat multiple-epitope gene was designed, which contained three copies of two epitopes (fragments A and B) and two copies of one epitope (fragment C) (named Polytope) (Figure 1). Then, the Polytope sequence was linked to the IL-2 and FcIgG sequences. To minimize interference between epitopes, linker sequences GG were used to separate epitopes. In this study, the Polytope-IL-2 gene was used to construct recombinant vaccines.

Tertiary structure of the recombinant Polytope protein in comparison with the natural VP1 protein of FMD virus and polytope-IL2 protein were predicted by Swiss Model software.

***PCR amplification***

Oligonucleotide primers with restriction sites at the 5´ ends (underlined) were designed using Primer Premier 5 (Premier Biosoft International) (Table1). The PCR amplification was carried out in total volume of 20 µl containing 2 μl of 4 mM MgCl_2_, 2 μl of 10X PCR buffer, 2 μl of 20 mM dNTPs, 1 μl of 10 pmol/μl of each primer, 1 μl of 50 ng/μl template DNA (pCU57-polytope-IL2/FcIgG), 1u/μl of *pfu* DNA polymerase and some deionized water up to a final volume of 20 µl. The PCR program to amplify polytope and IL-2 genes was performed with an initial denaturation step at 95 ^°^C for 3 min followed by 30 cycles of denaturation at 94 ^°^C for 1 min, annealing at 66 ^°^C for 30 sec and extension at 72 ^°^C for 30 sec, and a final extension at 72 ^°^C for 10 min. Also, the PCR program to amplify Polytope-IL-2 fusion gene was carried out as described below by using 94 ^°^C for 3 min, 35 cycles of 94 ^°^C for 45 sec, 62 ^°^C for 45 sec, and 72 ^°^C for 120 sec, and a final extension for 10 min at 72 ^°^C. The amplicons were purified and stored at -20 until use. 

***Construction of the recombinant plasmids***

pET32a (+) vector was double digestion by *EcoR*I and *Xoh*I enzymes. Purified fragments were ligated into pET32a through T4 DNA ligase procedure in 16 hr at 16 ^°^C. Recombinant pET32a/Polytope-IL2, pET32a/IL-2 and pET32a/Polytope plasmids were transformed to *Escherichia coli* strain DH5α cell and were grown overnight at 37 ^°^C on LB agar plates containing ampicillin (100 μg/ml). The recombinant expression plasmids were screened by PCR colony and confirmed by digestion with *EcoR*I and *Xoh*I enzymes. Also, the recombinant plasmids were sequenced using T7 universal primers.

***Protein expressions and purification***

For expression, the positive pET32(a)+ recombinant constructs were cultured in LB ampicillin medium. The recombinant vectors were induced with 1 mM isopropyl β-D-1-thiogalactopyranoside (IPTG) in a culture of bacteria with an optical density (OD)=0.6. Bacteria were incubated for 4 hr at 37 ^°^C with shaking at 240 rpm. Bacteria were then harvested by centrifugation (3000 g, 20 min, 4 ^°^C) and stored at -80 ^°^C. The pellet from a 100 ml bacterial culture was suspended in lysis buffer containing Tris 50 mM, ethylenediaminetetraacetic acid (EDTA) 5.0 mM, urea 8.0 M, at pH=8.0 and lysed by sonication. Cell lysate was subjected to centrifugation at 8000 g for 10 min at 4 ^°^C to separate the supernatant containing soluble materials from the pellet. Both the supernatant and the pellet were investigated to analyze protein expressions on SDS-PAGE 12%.

Purification of the expressed pET32a-Polytope-IL-2 and pET32a-Polytope proteins were performed by chromatography through Ni-agarose (Qiagen, Hilden, Germany) from the insoluble phase, according to the manufacturer’s protocol. Also, the recombinant pET32a-IL-2 protein was purified from the soluble phase, according to the manufacturer’s protocol. The concentration of each expressed protein was determined by Bradford assay (18). In order to identify the recombinant proteins, the purified proteins were analyzed by SDS-PAGE (12%) and dot-blotting analysis. 

For dot-blotting, 2 μg/ml of each induced protein and uninduced extract of the transformed bacteria were dot-blotted on nitrocellulose membranes. The membranes were immersed in 1% bovine serum albumin and were shaken in the incubator for 30 min at room temperature. After that, the membranes were washed with PBST (phosphate-buffered saline, 0.1% (v/v) Tween) for 5 min, and then they were immersed in anti-His_6_ antibody (Roche) diluted 1:2000 for 1 hr on shaking incubator at room temperature. After that, the nitrocellulose membranes were washed four times for 5 min each time in PBST, and were incubated with anti-mouse immunoglobulin G -horseradish peroxidase (IgG-HRP) -conjugate antibody (Sigma, USA) that was diluted 1:1000 for 1 hr on shaking incubator at room temperature. The membranes were washed four times for 5 min each time in PBST. Color development was observed by adding diaminobenzidine dissolved in PBS and H_2_O_2_. 

***Experimental groups of mice and immunization***

Six to eight week old female BALB/c mice (Razi Vaccine and Serum Research Institute, Mashhad, Iran) were randomly distributed into five experimental groups (6 mice/group). The mice were kept in conventional animal facilities and received water and food *ad libitum*. All animal care and procedures were in accordance with institutional policies for animal health and well-being. 

The BALB/c mice were immunized by intraperitoneally injection on days 7 and 14 with the recombinant vaccines. Experimental groups consisted of 5 groups: Polytope (100 μg), Polytope+IL-2 (50 μg+50 μg), Polytope-IL-2 (100 μg) and inactivated monovalent vaccine of FMD virus for serotype O (100 μl). Similarly, a group of mice received 100 μl of PBS -1X as a negative control. 

***Humoral immune response***

One week after the last immunization, mice were bled and serum was collected by centrifugation at 3000 g for 20 min. After that, the supernatant was stored at -80 ^°^C. The humoral immune response was measured by indirect ELISA assay. Briefly, 96-well plates were coated with 10 μg/ml of the epitope recombinant protein in PBS at 4 ºC overnight. Plates were followed by blocking with 5% non-fat dry milk in PBST 0.01% for 2 hr at 37 ^°^C. Then, 100 µl of the diluted serum (1:100) was added to the wells and incubated for 90 min at 37 ^°^C. Coated plates were washed five times and 100 µl of 1:10000 dilution of HRP-conjugated goat anti-mouse IgG (subtypes IgG1 and IgG2a) (Sigma, USA) were added to the wells and incubated for 90 min at 37 ^°^C. After extensive washing, the reaction was developed by adding 100 μl/well TMB (3, 3′, 5, 5′-tetramethylbenzidine) substrate. Color development was stopped by adding 50 μl/well 2N H_2_SO_4_ after 10 min of incubation of the plates in dark at room temperature. The OD was measured at 450 nm / 630 nm wavelength using an ELISA reader (Bio-Rad, USA). 

***Cellular immune response***

In order to investigate the cellular immune response against immune-dominant epitopes of FMD virus, mice were sacrificed one week after the final immunization. Spleen cells were homogenized in 10 ml PBS containing 5 mM EDTA on ice. The spleen cells were washed twice with PBS-EDTA and mononuclear cells were isolated. Splenocytes enriched in culture medium RPMI-1640 from mice (3×10^6^ cells) were coated in 24-well plates and stimulated with the epitope recombinant proteins (10 μg/ml). Plates were incubated for 3 days at 37 ^°^C in a humidified atmosphere, and 5% CO_2_. At the end of incubation, cell culture supernatants were harvested and centrifuged at 300 g for 10 min and stored at -80 ^°^C for cytokine measurement. Interferon gamma (IFN-γ), IL-10 and IL-4 were measured in cultured supernatants using commercial ELISA kits, according to the manufacturer’s instructions (R&D System, Quantikine, USA). The standard curve was made for each cytokine by ELISA reader (Bio-Rad, USA). The cytokine concentration values for each sample were given in pg/ml. 

***Statistical analysis***

Differences in humoral and cellular responses between groups were analyzed by one-way ANOVA, followed by Turkey’s multiple comparisons test analysis using GraphPad v6.07 software (GraphPad Software Inc., SanDiego, CA, USA) to detect any significant differences among groups of the immunized mice. *P*-values less than 0.05 were considered statistically significant. Values were expressed as mean± SD in the text and figures.

## Results

***The recombinant proteins (pET32a/*** **Polytope-IL-2** ***, pET32a/*** **Polytope** ***and pET32a/IL-2) were expressed in E. coli)***

**Figure 2:**
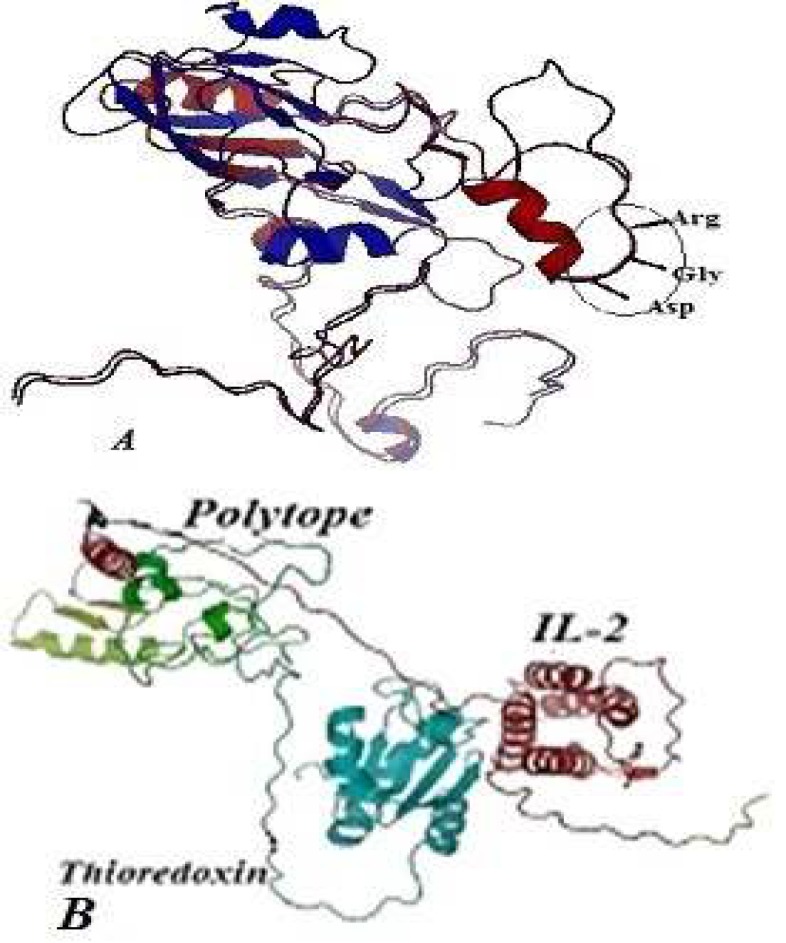
(A) Predicted 3D structure of the polytope protein in comparison with the natural VP1 protein of foot-and-mouth disease (FMD) virus. This figure showed the structure superimposition between the polytope protein and the VP1 of FMDV by PyMOL software. The natural VP1 of FMDV was shown by blue line and the polytope protein was shown by red line. (B) 3D structure of the polytope-IL-2 protein

Predicted 3D structure of the polytope protein in comparison with the natural VP1 protein of FMD virus and polytope-IL2 protein are shown in Figure 2. This figure showed the structure superimposition between the Polytope protein and the VP1 of FMD virus by PyMOL software. The loop-G-H region (amino acids 141-161) that plays an important role in binding the virus to the receptor is coincident in the recombinant and natural proteins. Also, the analysis showed that all predicted B and T-cell epitopes located on the outside of the VP1 antigen.

**Figure 3:**
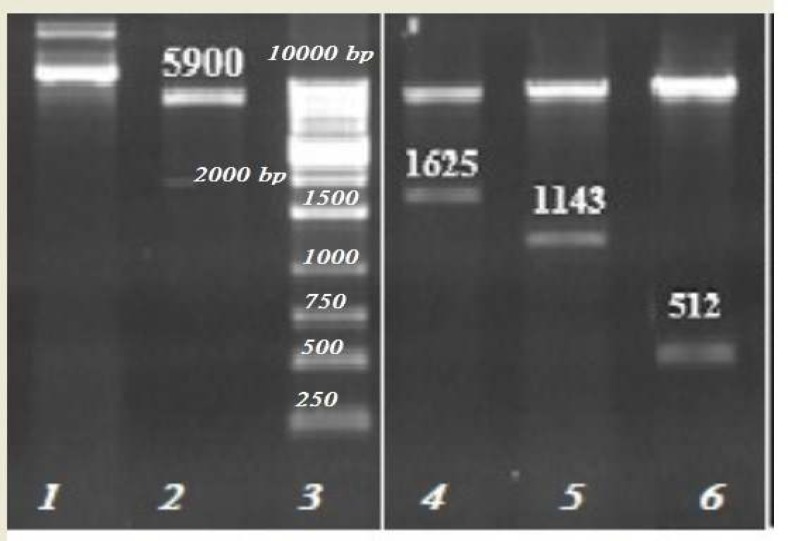
(A) Enzymatic digestion of the recombinant plasmids by EcoRI and XhoI (lane 1: undigested pET32a, lane 2: digested pET32a, lane 3: 1 kb DNA size marker, lane 4, 5 and 6: digested pET32/Polytope- interleukin-2 (IL-2), pET32/Polytope and pET32/IL-2 plasmids

Designed constructs of the polytope-IL-2, polytope and IL-2 were amplified using specific primers. The recombinant plasmids were transformed into DH5α. The integrity of the constructed recombinant plasmids was confirmed by restriction analysis with *EcoR*I and *Xoh*I enzymes. The existence of inserts into pET32a was confirmed by this method (Figure 3). The results of DNA sequencing of each construct showed that plasmids were correctly constructed with sequence integrity and right orientation. 

***Characterization of the expressed proteins by SDS-PAGE and dot-blotting analysis***

**Figure 4:**
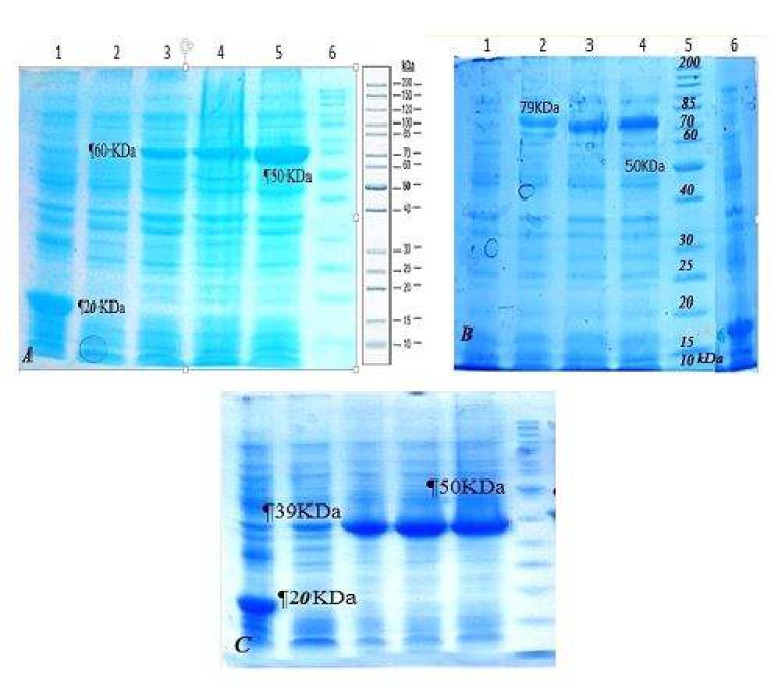
(A) SDS-PAGE analysis of the recombinant polytope protein. Lane 1, 2, 3, 4, 5 and 6: bacterial lysate expressing the induced pET32a, pET32a/polytope before the induction, pET32a/polytope protein in 1, 2 and 3 hr after induction and protein marker, respectively. (B) Analysis of the recombinant polytope- interleukin-2 (IL-2) protein. Lane 1, 2, 3, 4, 5 and 6: the bacterial lysate containing pET32a/polytope-IL-2 before the induction, pET32a/polytope-IL-2 protein in 1, 2 and 3 hr after induction, protein marker and induced pET32a, respectively. (C) Analysis of the recombinant IL-2 protein. Lane 1, 2, 3, 4, 5 and 6: the bacterial lysate containing pET32a/IL-2 before the induction, pET32a/IL-2 protein in 1, 2, 3 and 4 hr after induction and protein marker

The recombinant proteins were expressed after induction by IPTG. Proteins 79 kDa, 60 kDa and 39 kDa that belonged to the pET32a/Polytope-IL-2, pET32a/polytope and pET32a/IL-2, respectively, can be detected in Coomassie blue staining (Figure 4). The recombinant proteins were expressed in *E. coli *with peptide sequences fused with the 109 aa Trx•Tag™ thioredoxin protein and 6-histidine tag of pET32a (+). These additional amino acids increased the size of the expressed proteins about 20 kDa. 

**Figure 5:**
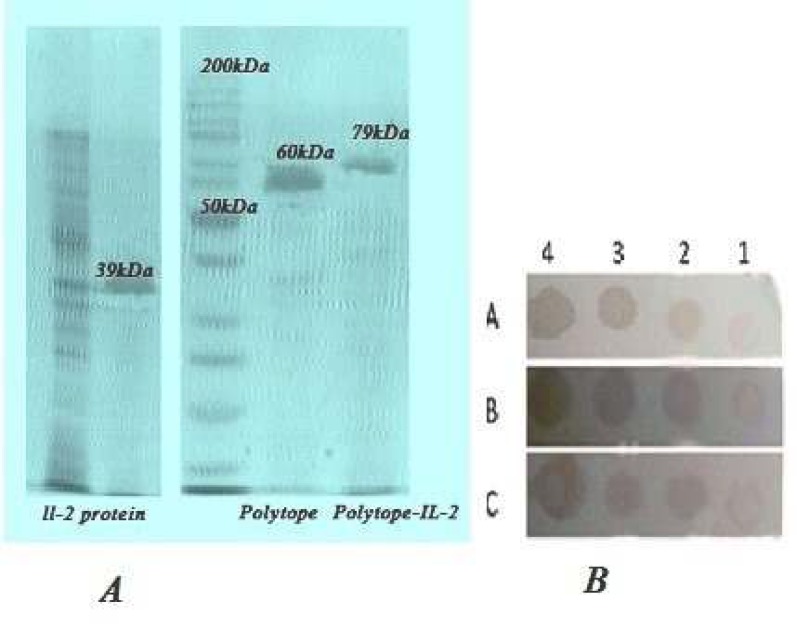
(A) SDS-PAGE analysis of the purified recombinant proteins including interleukin-2 (IL-2), polytope and polytope-IL-2. (B) Dot-blot analysis using anti-His tag antibody. Lane 1: extract of the transformed bacteria uninduced by isopropyl β-D-1-thiogalactopyranoside (IPTG), Lane 2, 3 and 4: the extract of transformed bacteria (*Escherichia coli*/pET32a/polytope-IL-2, *E. coli*/pET32a/Polytope and *E. coli*/pET32a/IL-2) induced by IPTG

The recombinant proteins were purified using Ni-NTA agarose column (Figure 5A). Using Bradford assay, the concentrations of recombinant Polytope-IL-2, Polytope and IL-2 proteins were determined 2.29, 2.84 and 3.02 mg/ml, respectively. The purified proteins were identified by SDSPAGE and dot-blotting analysis (Figure 5B). 

***Humoral response was induced using different vaccine combinations***

**Figure 6:**
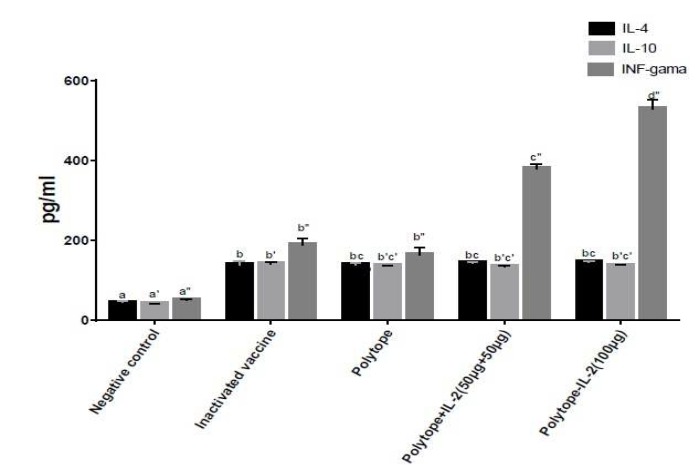
Antibody responses in the immunized mice. Data represent the mean±standard error of triplicates from 6 mice/group. Different letters refer to statistically significant differences between experimental groups (*P<* .05)

Humoral immune response was determined by measuring IgG antibodies using specific indirect ELISA in sera from the immunized and control mice. Results of total IgG, IGg1 and IgG2a showed that immunization using different treatments raised the level of antibody titer compared to the negative control group (*P*<0.05). Immunization with the recombinant polytope protein (50 μg)+IL-2 (50 μg) led to a strong specific total IgG and IgG1 responses higher than the other groups (Figure 6). The results of immunization with the recombinant polytope-IL-2 (100 μg) protein also showed a strong specific IgG2a response higher than the other groups. These results indicated that IL-2 could be recommended as a potential adjuvant for the VP1 protein and enhanced humoral immune response when co-expressed or co-inoculated with the VP1 protein of FMD virus. 

**Figure 7:**
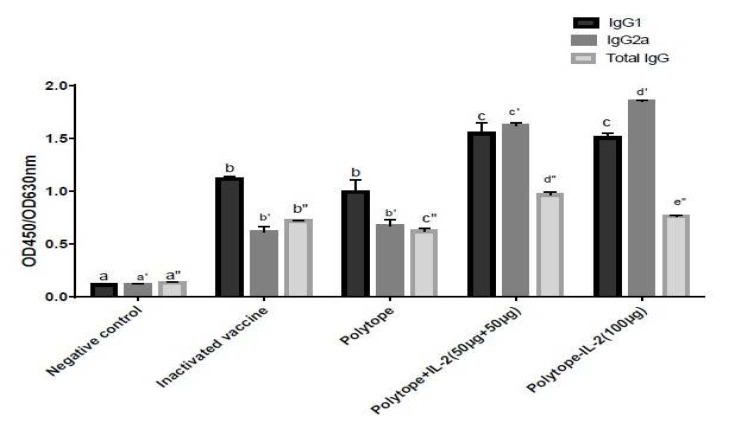
Cytokine responses in the mice immunized with the recombinant vaccines. The concentrations of each cytokine were determined (pg/ml) by ELISA. Data represent the mean±standard error of triplicates from 6 mice/group. Different letters indicate statistically significant differences between the experimental immunized groups (*P<* .05)

**Table 1:** Sequences of specific primers with restriction sites

Length (bp)	The amplified regions	primer sequences (5'-3')	Primer name and restriction enzymes
1155	Polytope	F-CCGGAATTCCGCGGGATCCGATACTTACCATG R-CCGCTCGAGGGCCAGCTGCATGCTATACATG	Epi – *EcoR*I Epi - *Xho*I
1637	Polytope-IL-2	F-CCGGAATTCCGCGGGATCCGATACTTACCATG R- CCGCTCGAGCGGGCTGGTGCTAATAATGCTCTG `	Epi-IL2-* EcoR*I Epi-IL2-* Xho*I
524	IL-2	F-CCGGAATTCATGTATAGCATGCAGCTGGCGAGCT R- CCGCTCGAGGTGCGGGCTGGTGCTAATAATGCT	IL2 – *EcoR*I IL2 - *Xho*I

***Cytokine response was induced using different vaccine combinations***

The secretion profiles of IFN-γ, IL-4 and IL-10 revealed that the epitope recombinant vaccines significantly stimulated the production of IFN-gamma, IL-4 and IL-10 in the spleen cells from the immunized mice but not from PBS-immunized mice (*P*<0.05). Also, the secretion profiles of IL-4 and IL-10 revealed that there were no significant difference between the mice immunized with the inactivated vaccine, Polytope, Polytope+IL-2 (50 µg+50 µg) and Poltope-IL-2 (100 µg) groups (*P*<0.05). The results of INF-gamma indicated that no significant difference was observed between the mice immunized with the inactivated vaccine and Polytope groups (*P*<0.05). Additionally, the production of IFN-γ in lymphocytes stimulated by the recombinant Polytope-IL-2 (100 µg) vaccine was significantly higher compared to the other groups (528.333 pg/ml). The maximum production of IL-4 was calculated in mice immunized by the recombinant Polytope-IL-2 (100 µg) vaccine (145 pg/ml). The production of IL-10 in lymphocytes stimulated by the inactivated vaccine was significantly higher in comparison with the other groups (141 pg/ml) (Figure 7). The minimum production of the IFN-γ (50.66 pg/ml), IL-4 (44 pg/ml) and IL-10 (40 pg/ml) were calculated in negative group (Figurer 7). The data showed that IL-2, as a molecular adjuvant for FMD virus vaccine, enhanced the levels of antibodies and also the secretion of IFN-γ, IL-4 and IL-10. It implies that IL-2, as a molecular adjuvant, could enhance the production of Th1 (IFN-γ) and Th2 (IL-4 and IL-10) cells when co-expressed or co-inoculated with the recombinant vaccines.

## Discussion

The results revealed the possibility of appropriate production of the recombinant polytope, polytope-IL-2 and IL-2 proteins. Our finding showed that the produced recombinant proteins were expressed at appropriate levels in prokaryote cell. These findings are in accordance with the results of other surveys that showed that *E. coli* could be used as a proper host to produce the recombinant VP1 protein (3, 14, 17, 19, 20). SDS-PAGE analysis revealed the presence of protein sizes of the purified recombinant proteins, which were in agreement with the theoretical prediction of molecular weights of the expressed proteins. Finding of this study was in agreement with the results of investigators that showed the protein VP1 of FMD virus is expressed in insoluble phase (21, 22).

 In order to evaluate whether IL-2 co-expressed or co-injected with the epitope recombinant protein of VP1 protein has the ability to stimulate humoral and cellular immune responses for FMD virus, we evaluated the secretion levels of three major cytokines. IFN-γ, an important components of Th1 immune responses, stimulates macrophages, increases the level of MHC class II and induces IL-12 secretion, which induces the differentiation of Th1 cells. Analysis of the mechanism of inhibition on FMD virus recommended that IFN-γ did not inhibit the viral replication through induction of nitric oxide, but continuous treatment with IFN-γ severely restricts FMD virus replication and even cures persistently infected bovine epithelial cells (23). According to our finding, using the Polytope co-expressed or co-injected with IL-2 as a molecular adjuvant could lead to Th1 immune response pattern. IL-4 and IL-10, as other component of cytotoxic immune response, stimulate Th2 immune responses. These cytokines are produced by various cells including monocytes, macrophages, B-lymphocytes, CD4 + T cells and CD8+T cells. In this study, secretion of IL-4 and IL-10 in lymphocyte cells from the mice immunized with the recombinant polytope-IL-2 vaccine and inactivated vaccine groups were higher than other groups, but no significant differences were found in comparison with other groups immunized with the recombinant vaccines. The lower ratio of IL-10 and IL-4 in comparison to INF-γ in this study indicated that the recombinant vaccines of VP1 protein induced cellular immune response through Th1 response pathway. The result demonstrated that although immuno-dominant epitopes of the VP1 protein (polytope) alone could induce immune response, but it revealed that using a suitable adjuvant coupled with the epitope recombinant proteins can improve vaccination efficiency and induce better immune responses (24). Therefore, the immuno-dominant epitopes of the VP1 protein from FMD virus that are co-expressed or co-injected with IL-2 enhance humoral and cellular immune responses. The results indicated that IL-2 could induce both humoral and cellular immune responses, suggesting that IL-2 could be used as potent adjuvant. The results indicated that Th1 and Th2 type cytokines were increased after co-inoculation with the recombinant vaccines. Shi *et al*. (2006) reported that although the VP1 protein of FMD virus alone could induce both humoral and cellular immune responses in mice, both humoral and cellular immune responses could be considerably enhanced when the mice were co-inoculated with the VP1 protein and bovine IFN-gamma (25). Yang *et al*. (2008) showed that antigenic epitopes of the protein VP1 of FMD virus induce not only humoral and cell-mediated immune responses but also confer full protection against FMDV in target animals (26). In a study, Du *et al*. (2008) constructed the recombinant adenovirus rAd-pIFNa-VP1 co-expressing VP1 and porcine interferon alpha as fusion protein, which could induce significantly higher humoral and cellular immune responses than co-administration of rAd-VP1 + rAd-pIFNa in mice and provide complete protection in guinea pigs (27).

This result is in accordance with prior immunization studies in mice, which revealed that the recombinant FMD virus vaccines could stimulate humoral and cellular responses (25- 28). Also, the result is in agreement with finding of similar studies that revealed that IL-2 or IL-1, as a molecular adjuvant, in combination with the VP1 protein of FMD virus improves the humoral and cellular responses (15, 29).

## Conclusion

We developed a recombinant polytope and polytope-IL-2 plasmids that were constructed with VP1 protein, which contained the critical epitopes for inducing an immune response to the FMD virus. According to our finding, the epitope recombinant plasmid of the VP1 protein co-expressed or co-inoculated with IL-2 was effective in inducing an enhanced immune response. The data of current study revealed that IL-2, as an adjuvant for epitope recombinant vaccines, could improve both humoral and cellular immune responses when co-expressed or co-inoculated with the VP1 protein of FMD virus. Therefore, IL-2 can be recommended as a potential adjuvant for recombinant FMD virus vaccines. 
